# Comparison of Intracardiac Echocardiography and Transesophageal Echocardiography for Image Guidance in Percutaneous Patent Foramen Ovale Closure

**DOI:** 10.3390/medicina56080401

**Published:** 2020-08-09

**Authors:** Jeonggeun Moon, Yeonjeong Park, Su Jung Park, Pyung Chun Oh, Albert Youngwoo Jang, Wook-Jin Chung, Woong Chol Kang

**Affiliations:** Division of Cardiology, Department of Internal Medicine, Gil Medical Center, Gachon University College of Medicine, Incheon 21927, Korea; jeff76@gilhospital.com (J.M.); pyj0222@gilhospital.com (Y.P.); image4631@gilhospital.com (S.J.P.); likemed@gilhospital.com (P.C.O.); cardio_gil@gilhospital.com (A.Y.J.); heart@gilhospital.com (W.-J.C.)

**Keywords:** patent foramen ovale, intracardiac echocardiography, transesophageal echocardiography.

## Abstract

*Background and Objectives:* Transesophageal echocardiography (TEE) guidance is the current gold standard for catheter-based procedures in the treatment of structural heart diseases. Intracardiac echocardiography (ICE), which can be performed under local anesthesia, has been recently introduced and is becoming more widely used. We aimed to compare the efficacy and safety of ICE and TEE in patent foramen ovale (PFO) device closure. *Materials and Methods:* All 74 patients with a history of cryptogenic stroke undergoing PFO closure for secondary prophylaxis were selected from our registry. Intraprocedural TEE was performed by echocardiographer-cardiologists with the patient under general anesthesia. Conversely, ICE was performed with the patient under local anesthesia. Baseline characteristics, procedural details, and immediate outcomes were compared between the TEE and ICE groups (*n* = 49 and *n* = 25, respectively). *Results:* Although patients in the ICE group were older (47 ± 10 vs. 57 ± 7 years, *p* < 0.001), sex and comorbidity variables were similar between the two groups. The degree of inducible right-to-left shunt via the PFO, assessed using preprocedural TEE, was also comparable. Notably, fluoroscopy time (22 ± 18 vs. 16 ± 7 min, *p* = 0.030), radiation dose (498 ± 880 vs. 196 ± 111 mGy, *p* = 0.022), and total procedural time in the catheter laboratory (99 ± 30 vs. 67 ± 12 min, *p* < 0.001) were significantly lower in the ICE group than those in the TEE group. The entire hospital stay was similar between groups (3.8 ± 2.2 vs. 3.4 ± 1.3 days, *p* = 0.433). No procedural complications, such as device embolization, pericardial hemorrhage, major bleeding, mortality, or access-related vascular injury were reported in either group. *Conclusions:* ICE-guided PFO device closure is quicker and less hazardous in terms of radiation exposure than the TEE-guided procedure, with similar procedural outcomes and duration of hospital stay.

## 1. Introduction

The foramen ovale is a physiological shunt of fetal circulation and normally fuses spontaneously after birth. However, the process of fusion fails in about a quarter of the general population, resulting in a patent foramen ovale (PFO) [1]. PFO is believed to be associated with cryptogenic stroke (CS) [2,3] because it could serve both as a nidus for in situ thrombus formation and a conduit of paradoxical embolism [4]. Patients with CS are usually young; hence, prevention of a recurrence is of paramount importance. With the recent introduction of sealing devices and advances in interventional techniques, PFO device closure is being performed more frequently, and it has shown better long-term outcomes than medical therapy in select patients with a history of CS [5,6,7].

PFO device closure is usually performed under the guidance of real-time transesophageal echocardiography (TEE) with the assistance of echocardiographer-cardiologists. However, the main drawback of TEE-guided PFO device closure is the requirement of general anesthesia, which necessitates an anesthesiology team. Alternatively, the recently introduced intracardiac echocardiography (ICE), which is usually performed by an interventional cardiologist, is being more widely used to guide the closure of interatrial communications because it is well tolerated by patients [8,9,10]. Although approximately half of the interatrial communication closures currently being performed are done under ICE guidance, no direct comparison has been reported between the conventional TEE-guided PFO closure and the newer ICE-guided procedure [8,9]. In this study, we aimed to compare the efficacy and safety of the two device closure procedures in patients with CS.

## 2. Materials and Methods

### 2.1. Study Population

This was a retrospective observational study using the PFO registry of the attending hospital. From March 2014 to September 2019, all 165 patients who had experienced a CS or recurrent transient ischemic attack (TIA) had a PFO documented using TEE, and underwent PFO device closure, were entered in the registry. Among them, 74 patients were identified as the study population.

CS was defined as a stroke with no identifiable cause after excluding carotid or intracranial artery stenosis, atrial fibrillation, intracardiac thrombus, and atheromatous plaque at the aortic arch [2]. PFO closure was determined according to the discretion of the heart team (consisting of an interventional cardiologist, an echocardiographer-cardiologist, a neurologist, and a radiologist) based on clinical data, echocardiographic findings, and patient preference. All patients (51 men, overall mean age 50 years) had a PFO of more than moderate grade, as assessed according to previous studies [11,12]. The Amplatzer^®^ PFO Occluder (St. Jude Medical, St. Paul, MN, USA), GORE^®^ Septal Occluder (WL Gore and Associates Inc., Newark, DE, USA), and Occlutech Figulla^®^ PFO Occluder (Occlutech GmbH, Jena, Germany) were implanted in all patients. The current study conformed to the Declaration of Helsinki (sixth revision). The Institutional Review Board of Gil Medical Center, Gachon University College of Medicine approved this study (GDIRB 2014-35, 24 February 2014), and all patients provided written informed consent before enrollment.

### 2.2. Assessment of PFO Shunt Grade and Presence of an Atrial Septal Aneurysm (ASA)

PFO shunt size was assessed by using TEE-based agitated saline contrast study with the Valsalva maneuver. A shunt was defined as the appearance of contrast bubbles in the left atrium (LA) within three cardiac cycles of opacification of the right atrium (RA). Shunt degrees were defined as mild, moderate, and severe if 3–9, 10–30, and >30 contrast bubbles, respectively, appeared in the LA [11,12,13]. Shunt at rest was defined as the appearance of contrast bubbles in the LA within three cardiac cycles of RA opacification with normal respiration or presence of shunt flow on color Doppler [11,12]. ASA was defined as interatrial septal excursion of ≥10 mm from the midline during the cardiac cycle [14].

### 2.3. TEE Guided PFO Closure Procedure

The closure procedure was performed under general anesthesia. All procedures were performed by one operator (W.C.K.). One experienced echocardiographer-cardiologist (J.M.) performed TEE to help the operator make decisions during the procedure. After achieving femoral venous access, the PFO was crossed with a 5-Fr multipurpose catheter, which was advanced into the left upper pulmonary vein and exchanged over a 0.035-inch J-tipped stiff guidewire for an 8- or 9-Fr guiding sheath. Procedural anticoagulation was initiated with intravenous administration of 5000 units of unfractionated heparin. Thereafter, additional heparin was administered throughout the procedure to maintain an activated clotting time of ≥250 s. The size of the device was based on the TEE measurements of the distance between the PFO and aortic root. Device implantation was performed using previously described methods [15]. TEE was used to facilitate the implantation of the device and to confirm its successful positioning.

### 2.4. ICE Guided PFO Closure Procedure

All ICE-guided PFO closure procedures were performed under local anesthesia without conscious sedation. All procedures were performed by one operator (W.C.K.). The right and left femoral veins were punctured to introduce an 8- or 9-Fr and 8.5-Fr sheaths for the PFO closure device and ICE catheter, respectively. The ICE catheter was positioned in the RA through the sheath and was maneuvered for optimal visualization of the PFO. The procedure did not differ from the TEE-guided one except that the device was deployed under the guidance of ICE images.

### 2.5. Postprocedural Management and Study Endpoint

The recommended anti-platelet therapy after the procedure included daily intake of both aspirin 100 mg and clopidogrel 75 mg for at least 6 months. Thereafter, further use of any blood thinners was left to the discretion of the attending physician. The Risk of Paradoxical Embolism (RoPE) scoring system—comprised of age, history of hypertension, diabetes mellitus, stroke/TIA, smoking status, and cortical infarct on imaging—was used to stratify patients with PFO and CS according to the probability that the latter was attributable to the former [16,17]. Continuous electrocardiogram monitoring was performed during the PFO closure, and a 12-lead electrocardiogram was obtained immediately, 12 h and 36 h after the index procedure. Procedural success was defined as a successful device implantation with no procedure-related complications or in-hospital or morbidity or mortality.

### 2.6. Statistical Analysis

Continuous data are presented as mean ± standard deviation. Discrete variables are presented as absolute value and percentage. Continuous variables were compared using a two-sample *t*-test, whereas categorical variables were compared using chi-square and Fisher’s exact tests. Analysis of primary endpoint longitudinal data was performed using Kaplan–Meier estimates with log-rank test. *p*-values of <0.05 were considered statistically significant. The analysis was performed using SPSS version 20 (SPSS Inc., Chicago, IL, USA).

## 3. Results

Baseline clinical and TEE characteristics are shown in Table 1. The number of patients was larger in the TEE group than that in the ICE group (49 vs. 25). ICE group patients were older and had a lower prevalence of dyslipidemia. The main indication for PFO was stroke rather than TIA. The RoPE score was lower in the ICE group. On preprocedural TEE, the degree of inducible right-to-left shunt via the PFO was similar between the two groups. Resting shunt and the presence of ASA, both considered components of high-risk PFO, were comparable between the two groups.

**Figure 1:**
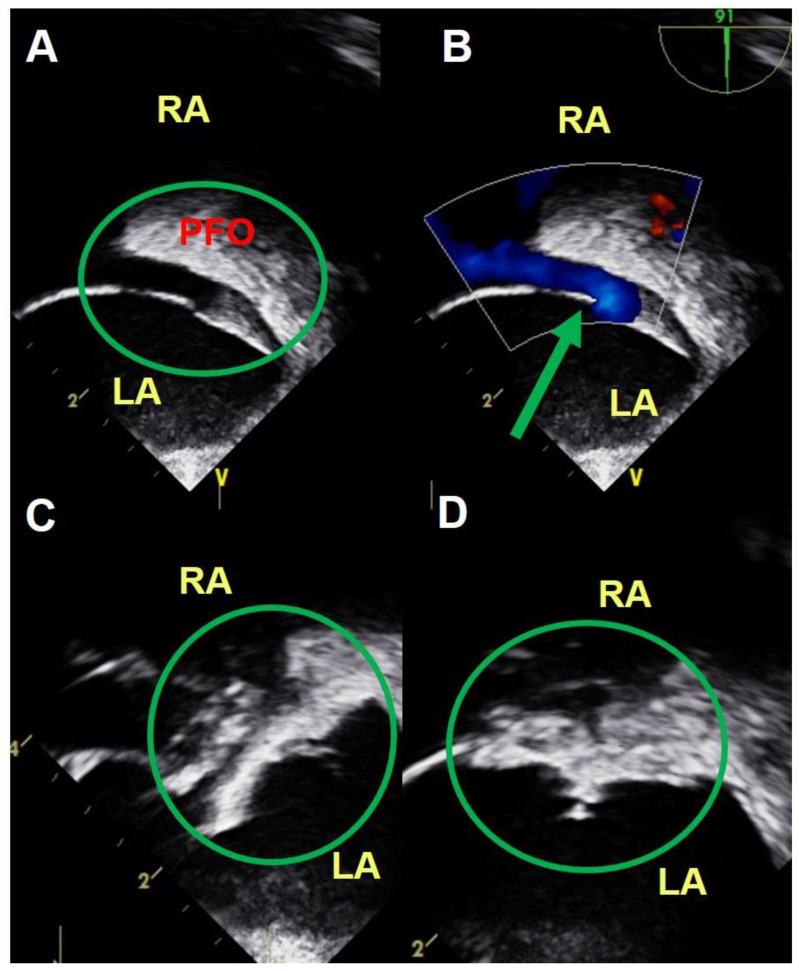
TEE-guided PFO device closure. A 40-year-old woman with a large PFO with resting left-to-right shunt underwent successful PFO device closure without any complication. TEE renders not only a two-dimensional PFO image (**A**) but also a good-quality color Doppler image of the resting left-to-right shunt via the PFO (**B**). An echocardiographer-cardiologist discusses with the operator about device deployment (**C**) and the result of the PFO device closure (**D**) during the procedure. TEE, transesophageal echocardiography; PFO, patent foramen ovale; RA, right atrium; LA, left atrium.

**Figure 2:**
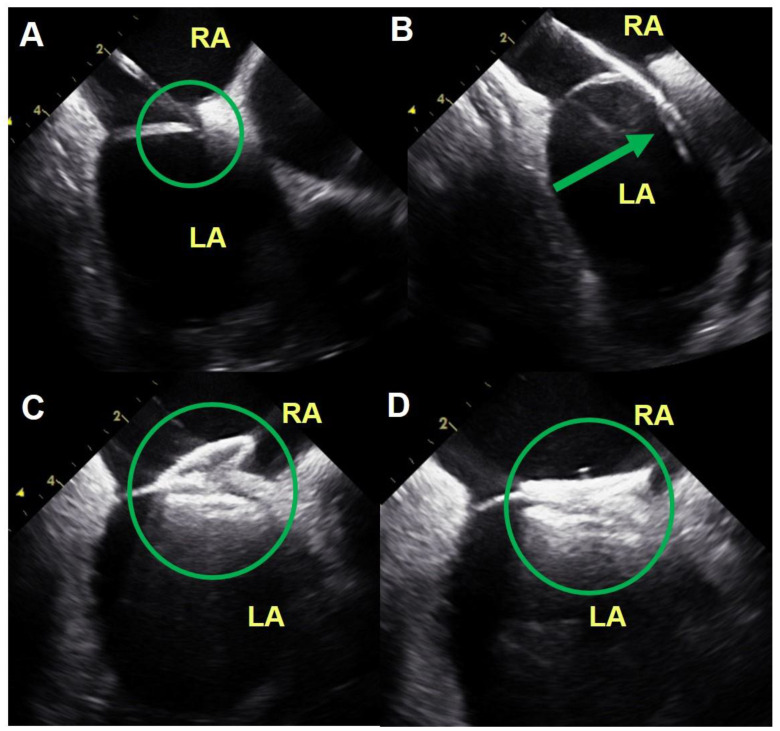
ICE-guided PFO device closure. The images were from a 68-year-old man who underwent successful PFO device closure without any complication ICE provides good images of the PFO inlet being pushed by a guiding catheter (**A**), passage of the catheter through the PFO (**B**), deployment of the PFO closure device (**C**), and good positioning of the device (**D**). ICE, intracardiac echocardiography; PFO, patent foramen ovale; RA, right atrium; LA, left atrium.

Figure 1 demonstrates a typical TEE-guided procedure. TEE renders not only a two-dimensional PFO image (A) but also a good-quality color Doppler image of the resting left-to-right shunt via the PFO (B). An echocardiographer-cardiologist discusses with the operator about device deployment (C) and the result of the PFO device closure (D) during the procedure. Figure 2 is a representative image of the ICE-guided procedure. ICE provides good images of the PFO inlet being pushed by a guiding catheter (A), passage of the catheter through the PFO (B), deployment of the PFO closure device (C), and good positioning of the device (D).

Table 2 provides the procedural and periprocedural details. Notably, fluoroscopy time and radiation dose were significantly lower in the ICE group than those in the TEE group. Total time in the catheterization laboratory was also notably shorter in the ICE group, and the same was true for the time from the venous puncture to vascular closure, time from arrival at the catheterization laboratory to venous puncture, and time from vascular closure until exiting the catheterization laboratory (*p* < 0.05 for all). The length of hospital stay did not differ between the TEE and ICE groups. No procedural complications were reported in both groups.

## 4. Discussion

The number of cases of PFO device closure for secondary prophylaxis of CS has been consistently increasing since its introduction in the late 1980s [18]. Most of the interventional procedures are performed with imaging guidance using TEE, which has been the modality of choice, or ICE, which was more recently introduced and is being increasingly used [19]. Although TEE is the diagnostic test of choice for preprocedural assessment of a PFO to define the anatomy of the atrial septum and its surrounding structures, intraprocedural TEE requires deep sedation, which is the main shortcoming of the semi-invasive imaging tool. Meanwhile, the strengths of the more recently introduced ICE include the following: (1) as patients normally tolerate ICE well, general anesthesia is scarcely needed; (2) ICE provides good image quality; and (3) ICE is performed by interventional cardiologists themselves [10]. Therefore, ICE-based PFO closure is expected to be superior to the TEE-guided procedure in terms of procedural time and logistics. In addition, ICE reduces the radiation hazard to both patients and interventionists by decreasing fluoroscopy time, and may improve outcomes in interventional procedures for treating arrhythmia [20,21] or structural heart disease [9,22]. In this study, we compared the procedural efficacy and safety of TEE-guided and ICE-guided PFO device closure and found equal procedural outcomes. Of note, as clearly proved in this study, using ICE in PFO device closure remarkably shortens the procedural time. ICE-guided procedure also lowers the requirement for fluoroscopic assessment during the intervention compared with TEE-guided procedure. To the best of our knowledge, this is the first study to directly compare TEE and ICE as guiding tools for PFO device closure.

The feasibility of ICE to guide device closure for intreratrial septal communication has been established [10,19,23,24]. However, ICE is not yet universally used in PFO device closure. The reasons for this include relative lack of experience of interventional cardiologists and the concern about potential complications such as vascular injury of femoral access site, cardiac perforation resulting in tamponade, and arrhythmia resulting from intracardiac stimulation by ICE probe navigation. Nevertheless, Alqahtani et al. [19] reported that ICE did not increase procedure-related complications when compared with TEE in guiding interatrial communication closure. The current study also demonstrated the safety of ICE-guided PFO closure in that no major complications were observed in both patient groups, which suggests that PFO device closure is essentially safe when performed in an experienced center. Another strength of the ICE-guided procedure is provision of a better image of the fossa ovalis and septum primum/secundum with the probe in the RA very close to the PFO (Figure 2). Thus, ICE allows for guiding and monitoring of the entire procedure by the operator himself/herself and provides better images of the atrial septal anatomy, catheter movement, and device deployment than TEE (Figure 1 and Figure 2).

Although ICE-guided PFO closure is safe and effective, another issue that needs to be considered is the validation of ICE images. Vigna et al. [25] reported a significant disagreement in the anatomical evaluation of the PFO between preprocedural TEE and intraprocedural ICE images, although the clinical implications of this remain unclear. However, it needs to be taken into consideration, especially among patients with complex anatomic variations of the fossa ovalis, such as in ASA, because the preprocedural evaluation of PFO is normally performed with TEE rather than ICE.

This study has all the inherent limitations of non-randomized single-center studies. The patient numbers and demographic features were not equal between the TEE and ICE groups. In addition, the sample size was small; hence, the difference in numbers between the two groups was not adjusted for. The use of closure devices from different manufacturers were not evenly distributed in the TEE- and ICE- group for technical issues and might be a confounder. Our institution is a tertiary referral medical center; hence, the current study patients may not be representative of all CS patients undergoing PFO device closure for secondary prevention. Notably, we enrolled consecutive patients, and the main operator in all procedures was identical (W.C.K, who has performed PFO-device closure in 165 patients over 6 years as of January 2020); therefore, the procedural learning curve favors a shorter procedural time in the ICE group because ICE was introduced later (June 2018) than TEE at our center. Atrial arrhythmia, including atrial premature complex, sustained-/non-sustained atrial tachycardia, or atrial fibrillation, occurs occasionally during PFO-device closure. Although no atrial fibrillation was detected in the current cohort, we do not have data of procedure-related transient cardiac rhythm disorder, so they were omitted from analysis.

## 5. Conclusions

ICE-guided PFO device closure is quicker and less hazardous in terms of radiation exposure than the TEE-guided procedure, with similar procedural outcomes and hospital stay duration. As such, ICE is a promising imaging modality for its safety and efficacy, and it would be more widely used to guide the PFO closure. However, the discrepancy between preprocedural TEE and intraprocedural ICE images requires validation.

## Figures and Tables

**Table 1:** Baseline clinical and transesophageal echocardiographic characteristics.

	TEE (*n* = 49)	ICE (*n* = 25)	*p*-Value *
*Clinical Characteristics*			
Age, years	47 ± 10	57 ± 7	<0.001
Female sex, *n*	16 (33%)	7 (28%)	0.683
Smoking, *n*	15 (31%)	11 (44%)	0.254
Body mass index, kg/m^2^ Comorbidities, *n*	24.9 ± 3.6	24.8 ± 3.2	0.943
Hypertension	15 (31%)	11 (44%)	0.254
Diabetes mellitus	7 (14%)	7 (28%)	0.154
Dyslipidemia	19 (39%)	3 (12%)	0.017
Coronary artery disease	1 (2%)	0 (0%)	0.472
Peripheral artery disease	1 (2%)	0 (0%)	0.472
Myocardial infarction	0 (0%)	0 (0%)	-
Heart failure	0 (0%)	0 (0%)	-
Chronic obstructive pulmonary disease	0 (0%)	0 (0%)	-
Indication for PFO closure, *n* (%)	-	-	-
Stroke	41 (84%)	24 (96%)	0.125
Recurrent TIA	8 (16%)	1 (4%)	0.125
Cortical infarct on imaging	32 (65%)	18 (72%)	0.073
RoPE score	6.4 ± 1.7	5.5 ± 1.4	0.042
*Preprocedural TEE Characteristics*			
Degree of shunt at Valsalva maneuver, *n* (%)	-	-	0.223
Mild	12 (24%)	4 (16%)	-
Moderate	18 (37%)	6 (24%)	-
Severe	19 (39%)	15 (60%)	-
Shunt at rest, *n* (%)	8 (16%)	4 (16%)	0.971
Atrial septal aneurysm, *n* (%)	0 (0%)	1 (4%)	0.159

* *p* < 0.05 was considered significant; TEE, transesophageal echocardiography; ICE, intracardiac echocardiography; PFO, patent foramen ovale; TIA, transient ischemic attack; RoPE, Risk of Paradoxical Embolism.

**Table 2:** Procedural and periprocedural details.

	TEE (*n* = 49)	ICE (*n* = 25)	*p*-Value *
Implanted device, *n* (%)	-	-	<0.001
Gore Septal Occluder	20 (41%)	0 (0%)	-
Amplatzer PFO Occluder	17 (35%)	0 (0%)	-
Occlutech PFO Occluder	12 (24%)	25 (100%)	-
Device size, *n* (%)	-	-	0.162
18 mm	5 (10%)	0 (0%)	-
25 mm	37 (76%)	23 (92%)	-
30 mm	7 (14%)	2 (8%)	-
Fluoroscopy time, min	22 ± 18	16 ± 7	0.030
Radiation dosage, mGy	498 ± 880	196 ± 111	0.022
Total time in cath. lab., min	99 ± 30	67 ± 12	<0.001
From venous puncture until vascular closure	53 ± 29	43 ± 11	0.020
From arrival at cath. lab. until venous puncture	23 ± 10	13 ± 4	<0.001
From vascular closure until exiting the cath. lab.	23 ± 8	11 ± 3	<0.001
Length of stay, days	3.8 ± 2.2	3.4 ± 1.6	0.433
Complication, *n* (%)	-	-	-
Atrial fibrillation	0 (0%)	0 (0%)	-
Device embolization	0 (0%)	0 (0%)	-
Pericardial effusion with tamponade	0 (0%)	0 (0%)	-
Major bleeding	0 (0%)	0 (0%)	-
Death	0 (0%)	0 (0%)	-
Access-related complications	0 (0%)	0 (0%)	-

* *p* < 0.05 was considered significant; TEE, transesophageal echocardiography; ICE, intracardiac echocardiography; PFO, patent foramen ovale; cath. lab., catheterization laboratory.
